# The increasing burden of pancreatic cancer in Brazil from 2000 to 2019: estimates from the Global Burden of Disease Study 2019.

**DOI:** 10.1590/0037-8682-0271-2021

**Published:** 2022-01-28

**Authors:** Diogo Oliveira Chaves, Aline Cândida Bastos, Alessandra Maciel Almeida, Maximiliano Ribeiro Guerra, Maria Teresa Bustamante Teixeira, Ana Paula Souto Melo, Valéria Maria de Azeredo Passos

**Affiliations:** 1 Faculdade Ciências Médicas de Minas Gerais, Programa de Pós-Graduação em Ciências da Saúde, Belo Horizonte, MG, Brasil.; 2 Universidade Federal de Juiz de Fora, Programa de Pós-Graduação em Saúde Coletiva, Juiz de Fora, MG, Brasil.; 3 Universidade Federal de São João Del Rey, Faculdade de Medicina, Divinópolis, MG, Brasil.; 4 Universidade Federal de Minas Gerais, Faculdade de Medicina, Belo Horizonte, MG, Brasil.

**Keywords:** Pancreatic câncer, Mortality, Incidence, Social inequality

## Abstract

**INTRODUCTION:**

Pancreatic cancer is increasing worldwide. The burden of pancreatic cancer in Brazil and its states was analyzed and compared with that from the USA and China.

**METHODS:**

This is a descriptive study of the incidence and mortality estimates from the Global Burden of Disease 2019 study, from 2000 to 2019. The Brazilian states presenting the highest and lowest socio-demographic index (SDI) were selected from each of the five regions. The SDI consists of the *per capita* income, education, and fertility rate of each population.

**RESULTS:**

A significant increase was found in age-standardized incidence and mortality of pancreatic cancer in all three countries, with differences in magnitude and annual increases. In Brazil, this incidence rose from 5.33 [95% Uncertainty Interval (UI): 5.06- 5.51] to 6.16 (95% UI: 5.68- 6.53) per 100,000 inhabitants. China and the Brazilian states with the lowest SDI, such as Pará and Maranhão, showed lower incidence and mortality rates, although presenting the highest annual increases. No difference was found between the sexes. A higher mortality rate was observed for those individuals of 70+ years, which was three to four times higher than those aged 50 to 69 years.

**CONCLUSIONS:**

The increasing burden of pancreatic cancer in the studied countries, and the higher estimates for the elderly in a fast-aging country such as Brazil, indicates that more resources and health policies will be necessary. The greatest increase in the states with lower SDI reflects inequalities in the access to diagnosis and registries of this cancer.

## INTRODUCTION

In Brazil, as well as in many countries around the world, mortality rates for pancreatic cancer have been increasing^1^ ^,^ ^2^. It is the most fatal of the main types of cancer, with an average time of survival of only about 6 months, with a 5-year survival rate of approximately 6%^3^ ^,^ ^4^ ^,^ ^5^. In 2014, the age-standardized mortality rates were 5.1 deaths/100,000 for men and 3.8 deaths/100,000 for women^6^. Pancreatic cancer accounted for 17.4% of all deaths by cancer, with age-standardized mortality rates of 5.0 and 5.5/100,000 inhabitants in 1990 and 2015, respectively^7^.

Incidence of hospitalization in the Brazilian Unified Health System (SUS, in Portuguese) for pancreatic cancer also increased. Between 2002 and 2015, an increase was reported in hospitalization rates, from 2.4 to 4.5/100,000 inhabitants, with an increase of 75% and 109% in each region of the country, mostly in state capitals^8^.

It is vital for health policymakers to understand the burden of pancreatic cancer in the country and its states. Decision-making is based on indicators for time and geographic comparison, and comparisons demand care in terms of the quality and comparability of data from different places and times. The homogeneity in the concepts of the causes of the disease was possible due to the choosing of the International Classification of Diseases (ICD). However, there is still a high level of heterogeneity in terms of degree of coverage and quality of information, which must be considered in the modeling of studies^9^.

The Global Burden of Disease (GBD) study proposes the use of standardized methodology, with correction of under-reporting and garbage codes, allowing for a comparison of the magnitude of the disease burden between countries and over time^10^. This study compares the Brazilian burden of pancreatic cancer with the burden of two countries that have also large populations: China, a middle-income country like Brazil, and the United States of America, a high-income country, from 2000 to 2019. Since 2015, the disease burden has also been estimated in all Brazilian states, thus allowing for a better understanding of regional disparities^10^. Consequently, this study also aimed to analyze the burden of pancreatic cancer among the states of Brazil during the same period.

## METHODS

This work is a descriptive study, analyzing the estimates of incidence, mortality, and years of life lost (YLL) for pancreatic cancer in Brazil and two counties used for comparison, according to place, sex, and age groups, between 2000 and 2019. All estimates were produced by the Institute for Health Metrics and Evaluation (IHME) and are available at the IHME website at: http://ghdx.healthdata.org/gbd-results-tool ^7^. 

The malignant neoplasm of the pancreas was defined according to the codes of the tenth revision of the ICD (ICD-10, C25 - C25.9)^7^.

The population-based cancer registries (RCBP, in Portuguese) were used as sources to calculate the incidence estimates^11^, while the mortality estimates used the Mortality Information System (SIM, in Portuguese), from the data of the Department of Information Technology of the Unified Health System (DATASUS, in Portuguese)^12^. To calculate rates, the population estimates provided by the Brazilian Institute of Geography and Statistics (IBGE, in Portuguese) were used as the denominator^13^. All Brazilian data sources are available at:http://ghdx.healthdata.org/gbd-2019/data-input^14^ ). 

Estimating mortality is the first step to GBD cancer estimation. Spatiotemporal Gaussian process regression was used to generate mortality estimates by all causes (mortality envelope) for 204 countries, including Brazil^15^. After correcting for the under-reporting of deaths for each Brazilian state, mortality according to sex and age is obtained, using a combination of life tables, death distribution methods, and regression techniques^7^ ^,^ ^15^. Once the mortality envelope has been obtained, with the total number of deaths per year, an estimation of specific mortality by cause of death is made. The cause of death ensemble modeling (CODEm) is a highly systematized tool to analyze the data concerning cause of death, using an ensemble of different modeling methods for rates, favoring those that perform best with the predictive validity testing. A new correction is then applied in this phase, with the distribution of causes with poorly specified causes of death, the garbage codes^7^ ^,^ ^14^ ^-^ ^15^. Age-standardized rates were used to compare populations with different age structures, in which the characteristics of the populations are statistically transformed to match those of the GBD world population standard^16^.

The burden of the disease is expressed by the YLL measurements, years lived with disability (YLD), and years lost to death or disability (DALY). The YLLs express the effect of premature deaths in the population and are obtained by multiplying the number of deaths of a given cause by the number of lost years at the age of each death, considering the highest life expectancy for that age group^7^. The Disease Modulation software (DisMod-MR) is a Bayesian meta-regression tool that enables the evaluation of all available data on incidence, prevalence, remission, and mortality for a disease. Additional information regarding incidence and mortality-incidence ratio estimation can be found in the specific publication on the global burden of cancer^16^. The YLDs are obtained by multiplying the prevalence of a sequela by the disability weight of the disease. DALY is obtained by adding the YLL and YLD measurements^14^ ^,^ ^16^. In this study, the YLL measurement represents the burden of pancreatic cancer, since the YLD is practically null.

For the analysis at a sub-national level, two states from each region of Brazil (North, Northwest, Midwest, Southeast, and South) were selected based on the Social Demographic Index (SDI). The SDI is obtained from the geometric mean of the fertility rate before 25 years of age, *per capita* income, and average education of the population above 15 years of age, for each population. Its values range from 0 (lowest) to 1 (highest)^17^. Brazil was classified in the average SDI category, with distinct values for each state. For this study, states with the highest and lowest SDI for each region of the country in 2019 were selected^17^.

For analysis at an international level, China and the USA were chosen, two countries with large populations like Brazil, but showing different levels of socioeconomic development (middle-income x high-income) and with a growing number of deaths caused by pancreatic cancer^18^ ^,^ ^19^. Moreover, the countries were chosen from among the locations in which the GBD 2019 study provided subnational estimates^14^.

For changes over time, annual rates of change are calculated as the difference in the natural log of the values at the start and end of the time interval^14^. The average annual proportions of change (Δ aa %) are presented in the tables.

Every estimate is expressed with its respective 95% uncertainty intervals (95% UI), which takes into consideration the errors that might have occurred in the modelling and reflects the uncertainty associated with the size of the samples used as data sources, the adjustments in the data sources to estimate mortality by all causes, as well as uncertainties in the estimation of model parameters and of model specifications of specific causes and all causes^20^. For that, 1,000 samples were produced of all measurements for every analytical phase^14^. The differences between estimates were considered statistically significant only if the estimates did not present a coincidence of the 95% UI. 

GBD 2019 analyses were conducted using Python, version 3.6.2; Stata, version 13; and R, version 3.5.0, and the statistical codeused for estimation is available online^14^. Further methodological details are also available^7^ ^,^ ^10^ ^,^ ^14^ ^-^ ^16^. 

This study was performed exclusively with public access data, with no identification of the subjects nor the need for free and informed consent. GBD-Brazil study was approved by the Research Ethics Committee of the Federal University of Minas Gerais (UFMG, CAAE Project number - 62803316.7.0000.5149). The authors have no conflicts of interest to declare.

## RESULTS

Table 1 shows the incidence of pancreatic cancer in the three countries (Brazil, China, and the USA) between 2000 and 2019, for both sexes. In Brazil, the age-standardized incidence rose from 5.33 (95% UI: 5.06-5.51) to 6.16 (95% UI: 5.68-6.53) per 100,000 inhabitants. These rates are similar with those in China, but twice as low as those in the USA. However, when the rates of annual increase are considered, between 2010 and 2019, a higher annual increase was found in China (0.82%) than in Brazil (0.19%) and in the USA (0.15%).

**TABLE 1:** Age-standardized incidence of pancreatic cancer in Brazil, China, the United States, and Brazilian States, between 2000 and 2019.

Countries (SDI 2019)	Population (2019)	Incidence per 100,000 inhabitants (95% UI)*			
		2000	2010	2019	∆ aa (%)**
					2010-2019
China (0.71)	1,402,509,320	3.86	5.20	5.78	0.11
		(3.59- 4.17)	(4.71- 5.69)	(4.94- 6.69)	
USA (0.87)	329,634,908	9.47	10.01	10.37	0.04
		(9.09- 9.68	(9.5- 10.3)	(8.94-11.96)	
Brazil (0.66)	211,755,692	5.33	5.71	6.16	0.08
		(5.06- 5.51)	(5.38- 5.91)	(5.68- 6.53)	
**Brazil Regions and States (SDI 2019)**					
**North**					
Pará (0.57)	8,690,745	3.4	3.75	4.00	0.07
		(3.13- 4.01)	(3.29- 4.22)	(3.47- 4.55)	
Amapá (0.65)	7,861,773	4.68	4.35	5.36	0.23
		(4.25- 5.1)	(3.97- 4.73)	(4.7- 6)	
**Northeast**					
Maranhão (0.50)	7,114,598	2.38	3.28	4.27	0.30
		(2-2.8)	(2.81- 3.75)	(3.57- 5.02)	
Rio Grande do Norte (0.60)	3,534,165	3.98	4.45	5.16	0.16
		(3.55-4.49)	(3.93-5.01)	(4.31-6.09)	
**Midwest**					
Goiás (0.65)	7,113,540	4.73	5.00	5.63	0.13
		(4.2-5.31)	(4.47-5.67)	(4.77- 6.59)	
Federal District (0.79)	3,055,149	6.83	6.75	6.66	-0.01
		(6.25- 7.41)	(6.18-7.27)	(5.79- 7.56)	
**Southeast**					
Minas Gerais (0.66)	21,292,666	5.04	5.56	5.64	0.01
		(4.69- 5.43)	(5.11- 5.97)	(4.9-6.37)	
São Paulo (0.72)	46,289,333	6.71	6.42	6.85	0.07
		(6.34- 7.03)	(6.04- 6.71)	(6.04- 7.64)	
**South**					
Paraná (0.68)	11,516,840	6.26	6.84	7.12	0.04
		(5.89-6.64)	(6.34- 7.28)	(6.23- 7.98)	
Santa Catarina (0.70)	7,252,502	7.28	6.95	7.34	0.19
		(6.79-7.8)	(6.42- 7.42)	(6.49- 8.26)	

**Source:** IHME, http://ghdx.healthdata.org/gbd-results-tool

*UI: Uncertainty Interval, **∆ aa: average annual change.

A significant increase in incidence can be seen in the states of the North, Northeast, and Southeast regions, and stability in the rates in the states of the Midwest and South regions. The higher the SDI, the higher the incidence of the disease. The states with the highest SDI, such as São Paulo, the Federal District, and Santa Catarina, show rates twice or three times as high as the state with the lowest SDI, Maranhão, which is lower than 3.0/100,000 inhabitants, considering age-standardized rates in each state. However, the states with the lowest SDI show a higher annual increase in incidence of pancreatic cancer, especially in the more recent period of 2010 to 2019, except for Minas Gerais, which showed a low annual increase (0.01%), Paraná with 0.04% and Pará with 0.07%, between 2010 and 2019 (Table 1).

Concerning the mortality for pancreatic cancer in Brazil, the age-standardized rates increased from 5.06 % (95% UI: 5.3-5.81) to 6.45 (95% UI: 5.94-6.84) per 100.000 inhabitants between 2000 and 2019. These rates are higher than those in China, but lower than those in the USA. However, the increase in mortality was faster in China, with annual increases of 0.63% between 1990 and 2010 and 0.10% between 2010 and 2019 (Table 2).

Much like the incidence rates, the mortality rates become higher with the increase in SDI. In the states with the lowest SDI, such as Pará (SDI=0.57) and Maranhão (SDI=0.50), the rates are half as high as in the states with higher SDI, like São Paulo, Paraná, Santa Catarina, and the Federal District, especially between 2010 and 2019. The states with the lowest SDI, like Maranhão and Goiás, show the highest annual increase in mortality. Maranhão (SDI=0.50) showed annual increases of 0.32 % and 0.36%, while Goiás (SDI= 0.65) showed annual increases of -0.01% and 0.12% between 1990-2010 and 2010-2019, respectively (Table 2).

**TABLE 2:** Age-standardized mortality due to pancreatic cancer in Brazil, China, the United States, and Brazilian States, between 2000 and 2019.

Countries (SDI 2019)	Mortality per 100,000 inhabitants (95% UI*)				
	2000	2010	2019	∆ aa** (%)	∆ aa (%)
				1990-2010	2010-2019
China (0.71)	4.07	5.43	5.99	0.63	0.10
	(3.76-4.46)	(4.95-5.91)	(5.12-6.93)		
USA (0.87)	9.16	9.67	10.06	0.10	0.04
	(8.75-9.38)	(9.15-9.98)	(9.43-10.52)		
Brazil (0.66)	5.6	5.98	6.45	0.09	0.08
	(5.3-5.81)	(5.61-6.22)	(5.94-6.84)		
**Brazilian Regions and States**					
**North**					
Pará (0.57)	3.73	3.93	4.18	0.06	0.06
	(3.28-4.2)	(3.42-4.41)	(3.62-4.74)		
Amapá (0.65)	5.09	4.57	5.64	0.15	0.23
	(4.59-5.55)	(4.11- 5)	(4.92-6.32)		
**Northeast**					
Maranhão (0.50)	2.6	3.46	4.7	0.32	0.36
	(2.15-3.14)	(2.97-3.99)	(3.96- 5.55)		
Rio Grande do Norte	4.18	4.65	5.38	0.27	0.16
(0.60)	(3.65-4,7)	(4.05- 5.24)	(4.5- 6.37)		
**Midwest**					
Goiás (0.65)	4.98	5.24	5.87	-0.01	0.12
	(4.42-5.58)	(4.67-5.87)	(4.94-6.96)		
Federal District (0.79)	7.36	.,27	7.16	-0.07	-0.01
	(6.7-8.01)	(6.63-7.87)	(6.15-8.19)		
**Southeast**					
Minas Gerais (0.66)	5.29	5.81	5.84	0.05	0.01
	(4.87-5.69)	(5.3- 6.23)	(5.11-6.58)		
São Paulo (0.72)	7.1	6.73	7.17	-0.03	0.06
	(6.64-7.47)	(6.28-7.07)	(6.35-7.98)		
**South**					
Paraná (0.68)	6.65	7.2	7.49	0.09	0.04
	(6.24- 7.07)	(6.64-7.7)	(6.52- 8.47)		
Santa Catarina (0.70)	7.75	7.29	7.67	0.03	0.05
	(7.18- 8.28)	(6.7-7.81)	(6.78- 8.65)		

**Source:** IHME, http://ghdx.healthdata.org/gbd-results-tool

*UI: Uncertainty Interval, **∆ aa: average annual change.

An increase in the burden of YLL was also observed in Brazil, China, and the USA. In Brazil, the rates of YLL per 100.000 inhabitants for pancreatic cancer increased, from 124.54 (95% UI: 119.99-128.11), in 2000, to 131.89 (95%: 126.36-135.84), in 2010, and to 140.00 (95% UI:130.85-147.71), in 2019. Among Brazilian states, there was a discrete increase in the YLL rates per 100,000 inhabitants in the states with the lowest SDI, identified only in the period between 2010 and 2019: Pará [89.44 (95% UI: 78.35-101.07) and 93.60 (95% UI: 81.48-106.21) ], Amapá [99.37 (95% UI: 91.38-107.69) and 122.53 (95% UI: 108.25-136.79) ], Maranhão [79.38 (95% UI: 67.64-92.4) and 104.5 (95% UI: 86.5-124.48) ], and Rio Grande do Norte [103.3 (95% UI: 80.87-116.33) and 118.88 (95% UI: 99.48-140.38) ]. The states of Minas Gerais and the Federal District showed a reduction in the YLL between 2010 and 2019 (Table 3).

**TABLE 3:** Age-standardized YLL due to pancreatic cancer in Brazil and compared places, between 2000 and 2019.

Countries (SDI 2019)	YLL per 100,000 inhabitants (95% UI*)		
	2000	2010	2019
China (0.71)	94.99	123.35	135.38
	(87.59- 103.63)	(112.22- 135.16)	(114.5- 157.74)
USA (0.87)	196.6	204.01	210,.9
	(191.52- 199.76)	(197.58- 208.34)	(201.14- 218.19)
Brazil (0.66)	124.54	131,89	140.00
	(119.99- 128.11)	(126.36- 135.84)	(130.85- 147.71)
**Brazilian Regions and States**			
**North**			
Pará (0.57)	84.21	89.44	93.60
	(74.16- 94.88)	(78.35- 101.07)	(81.48-106.21)
Amapá (0.65)	104.76	99.37	122.53
	(96.06- 113.93)	(91.38- 107.69)	(108.25- 136.79)
**Northeast**			
Maranhão (0.50)	60.87	79.38	104.5
	(50.49- 73.53)	(67.64- 92.4)	(86.5- 124.48)
Rio Grande do Norte (0.60)	94.22	103.3	118.88
	(82.8- 105.43)	(90.87- 116.33)	(99.48- 140.38)
**Midwest**			
Goiás (0.65)	109.49	115.70	130.22
	(87.22- 123.3)	(102.83- 129.81)	(109.35- 155.62)
Federal District (0.79)	149.00	140.58	135.2
	(137.81- 160.61)	(130.7-150.22)	(117.47-156.33)
**Southeast**			
Minas Gerais (0.66)	118.16	129.02	128.41
	(109.98- 126,5)	(120.22- 137.7)	(113.89- 144.04)
São Paulo (0.72)	153.49	14591	152.88
	(146.44- 160.2)	(138.27- 152.03)	(136.43- 169.42)
**South**			
Paraná (0.68)	143.83	157.29	161.06
	(136,31- 151,62)	(147,4- 166,8)	(141,06- 183,05)
Santa Catarina (0.70)	149	156.77	163.08
	(137.81- 160.61)	(146.68- 166.83)	(145.4- 184.7)

Source: IHME, http://ghdx.healthdata.org/gbd-results-tool

*UI: Uncertainty Interval.

When the risk of death by pancreatic cancer was investigated by sex and age group in 2019, similar mortality rates were found for both sexes. There was an increase with age in every state, with differences only in the magnitude of the rates. For instance, Maranhão (SDI=0.50) had rates of 0.69 (95% UI: 0.49-0.92) and 0.57 (95% UI 0.40-0.76) per 100,000 inhabitants for those between 15 and 49 years of age, of 14.11 (95% UI: 10.26-18.31) and 10.25 (95% UI: 7.66-13.41) per 100,000 inhabitants for those between 50 and 69 years of age, and of 44.43 (95% UI: 35.08-54.76) and 37.01 (95% UI: 29.95-44.88) per 100,000 inhabitants for those aged 70 years and over, for both men and women, respectively. The lowest rates were observed in São Paulo for the age group of 15 to 49 years of age, recording 1.12 (95% UI 1.37-0.90) for men, and 0.83 (95% UI 0.67-1.01) in the state of Santa Catarina for the age group of 50 to 69 years of age, with a rate of 21.53 (95% UI 17.68-26.08) for men. In the state of Paraná, for women, this rate was of 15.81 (95% UI 13.01-18.84). For those aged 70 years and over, the highest rates were in Santa Catarina, with a rate of 70.07 for men (95% UI 58.62-83.71) and of 67.73 (95% UI 55.67-80.05) for women (Table 4).

**TABLE 4:** Mortality rates by gender and age group in the states of Brazil, in 2019.

Region/States (SDI2019)	Mortality rate per 100,000 inhabitants (95% UI*)					
	Men			Women		
	(age groups in years)			(age groups in years)		
**North**	**15-49**	**50-69**	**70+**	**15-49**	**50-69**	**70+**
Pará (0.57)	0.64	12.14	37.27	0.47	8.76	35.12
	(0.52-0.79)	(9.91-14.85)	(30.73-44.29)	(0.36- 0.58)	(7.17-10.63)	(28. 26-42.14)
Amapá (0.65)	0.81	14.32	51.14	0.57	11.51	47.98
	(0.66-0.96)	(11.91-16.86)	(42.12-60.77)	(0.47-0.70)	(9.55-13.48)	(38.15-58.01)
**Northeast**						
Maranhão (0.50)	0.69	14.11	44.43	0.57	10.25	37.01
	(0.49-0.92)	(10.26- 18.31)	(35.08-54.76)	(0.40-0.76)	(7.66-13.41)	(29.95- 44.88)
Rio Grande do Norte (0.60)	0.99	15.01	48.34	0.64	11.29	47.75
	(0.76-1.31)	(11.35-19.38)	(37.84-61.33)	(0.47-0.86)	(8.7- 14.44)	(37.06- 59.70)
**Midwest**						
Goiás (0.65)	1.06	17.44	49.43	0.70	12.29	50.21
	(0.80-1.38)	(13.28- 22.49)	(39.70 - 61.49)	(0.51- 0.91)	(9.54-15.50)	(40.26- 61.81)
Federal District (0.79)	0.88	14.77	64.02	0.66	11.64	62.93
	(0.68-1.11)	(11.88- 18.35)	(53.02- 77.64)	(0.51-0.83)	(9.36-14.35)	(50.70- 77.00)
**Southeast**						
Minas Gerais (0.66)	1.05	16.68	49.83	0.76	12.78	53.59
	(0.86-1.29)	(13.80- 19.91)	(41.75- 59.06)	(0,61-0,93)	(10.56-15.27)	(43.35- 63.72)
São Paulo (0.72)	1.12	20.41	62.61	0.83	15.39	64.86
	(1.37-0.90)	(16.92- 24.07)	(52.28- 72.96)	(0.67- 1.01	(12.78-18.06)	(53.44-75.80)
**South**						
Paraná (0.68)	1.06	21.47	66,45	0.83	15.81	66.15
	(0.84-1.31)	(17.25- 25.98)	(54.19-78.38)	(0.66-1.05)	(13.01-18.88)	(54.52-78.82)
Santa Catarina (0.70)	1.06	21.53	70.07	0.83	15.65	67. 73
	(0.84-1.31)	(17.68-26.08)	(58.62- 83.71)	0.67- 1.03)	(13.00-18.84)	(55.67- 80.05)

**Source:** IHME, http://ghdx.healthdata.org/gbd-results-tool

*UI: Uncertainty Interval.

## DISCUSSION

The results of this study, using standardized methodology, converge with the findings of studies using other methodologies, which show a tendency for an increase in the burden of pancreatic cancer in Brazil and worldwide^1^ ^-^ ^3^ ^,^ ^6^ ^,^ ^18^ ^-^ ^19^ ^,^ ^21^ ^-^ ^23^. Moreover, this study described an evident influence of the level of social development in the magnitude and the annual growth of the rates of incidence and mortality in Brazil, in its states, as well as in the comparator countries.

The incidence and mortality rates in Brazil are similar with those of China, a middle-income country like Brazil, but lower than those of the USA, a high-income country. Even though China is the country which shows the lowest rates, it is also the country with the highest rates of annual increase throughout the period. Improvements in the socioeconomic situation of the country may have contributed to the fast growth and the proportional contribution of China to the global rates of pancreatic cancer. The lack of an efficient tracking program, the delay in early detection, and the low investments by the government, as compared to other types of cancer, were responsible for the lowest rates of diagnosis and treatment of pancreatic cancer in previous years, with a more recent increase in these indicators^18^. Those same causes can be identified as determining factors for the behavior of the burden of this cancer among the states of Brazil: those states with the highest SDI show higher rates, while the states with the lowest SDI, from the North and Northeast regions, show a higher annual increase, especially between 2010 and 2019. Projections of mortality by pancreatic cancer in Brazil also indicate that, in the period of 2015 to 2019, there will be an increase in mortality for men in the Northeast region^6^.

It is interesting to note that the number of YLLs for pancreatic cancer shows a smaller variation than the mortality rates throughout the studied period, with a small increase in China and Brazil and stability in the USA. Moreover, this only increased in the Brazilian states with a lower SDI, especially between 2010 and 2019. This may well be explained by the higher incidence of this type of cancer among the elderly, with a lesser impact of this metric even when there is an increase in cases or in diagnoses.

This study’s strength lies in the analysis of the annual growth rates of pancreatic cancer during the period. Although China and the Brazilian states with the lowest SDI show the lowest rates, they also show the highest annual growth of incidence and mortality. The higher the socioeconomic development, the higher the rates of incidence and mortality for pancreatic cancer. This phenomenon can be the result of improved access to the diagnosis of pancreatic cancer in these places^6^ ^,^ ^18^ ^,^ ^19^.

Less access to diagnosis can explain the lower rates in places with a lower SDI: Maranhão (SDI=0.51), which showed rates of age-standardized incidence of about half of those from São Paulo (SDI=0.72) and Paraná (SDI=0.68). A major difference was also observed in the mortality rates. For instance, in 2019, the rate varied from 3.68 (95% UI 3.38-4.00) in Maranhão to 6.65 (95% UI 6.34-6.97) in São Paulo and 6.80 (6.46-7.17) in Paraná. 

It is unlikely that, in the studied period, the increase in lethality will explain the increase in mortality for pancreatic cancer. On the contrary, one can expect that a disease with such a high lethality will show a reduction due to the scientific efforts to obtain new treatments and the possibilities of early diagnosis^21^.

Exposure to risk factors can also explain the differences in incidence and mortality for pancreatic cancer. As in other countries, only minor differences were observed in either incidence or mortality by sex, as compared to a major influence of age: pancreatic cancer is more common in people of over 50 years of age and more frequent in people over 70 years of age^22^ ^,^ ^23^. Other risk factors are also relevant, but they were not within this study’s scope of investigation. Obesity seems to increase the risk of pancreatic cancer due to chronic inflammation, mediated by adipocytes and immunosuppressant environments^24^. Smoking can also be contributed to the increases and differences in incidence rates in the studied countries^25^.

One additional strength of this study relies in the public availability of the estimation of the burden of disease, as well as the transparence of all data sources and modeling strategies used to obtain the estimates, as GBD 2019 complies with the Guidelines for Accurate and Transparent Health Estimates Reporting (GATHER) statement^14^. This is particularly important in pancreatic cancer estimation in Brazil, especially considering the paucity of epidemiological studies in the country. However, one likely limitation of this study refers to the quality of data sources in Brazil. Although the SIM may be considered a good quality source, and there are reports of good reliability of data comparisons between SIM and RCBP, differences can be expected between states in terms of coverage and quality of data^9^ ^,^ ^26^. 

In conclusion, the increase in the age-standardized rates suggests that the aging of the population could not explain this phenomenon, and further investigation is necessary to understand it. The countries are facing the growth in incidence and mortality by a highly lethal disease and with no method of tracking or early diagnosis currently available. The expected increase in the states with the worst access to health shows that Brazil needs to improve its universal health system, with funding and planning aimed at reducing inequalities, in terms of health risks and in access to treatment. The measures of fiscal austerity implemented since 2016 may represent a risk of making disparities between regions even worse^27^. It is also important for the country to invest in prevention measures aimed at the main risk factors, which are also common to other chronic non-communicable diseases^28^.
